# New Agents Are Coming, and So Is the Resistance

**DOI:** 10.3390/antibiotics13070648

**Published:** 2024-07-13

**Authors:** J. Myles Keck, Alina Viteri, Jacob Schultz, Rebecca Fong, Charles Whitman, Madeline Poush, Marlee Martin

**Affiliations:** 1 Department of Pharmacy, University of Arkansas for Medical Sciences, Little Rock, AR 72205, USA; 2 Independent Researcher, Thonotosassa, FL 33592, USA; 3 Department of Pharmacy, Central Arkansas Veterans Healthcare System, Little Rock, AR 72205, USA

**Keywords:** antimicrobial resistance, antimicrobial stewardship, infectious diseases, multi-drug resistance, global health, antibiotics

## Abstract

Antimicrobial resistance is a global threat that requires urgent attention to slow the spread of resistant pathogens. The United States Centers for Disease Control and Prevention (CDC) has emphasized clinician-driven antimicrobial stewardship approaches including the reporting and proper documentation of antimicrobial usage and resistance. Additional efforts have targeted the development of new antimicrobial agents, but narrow profit margins have hindered manufacturers from investing in novel antimicrobials for clinical use and therefore the production of new antibiotics has decreased. In order to combat this, both antimicrobial drug discovery processes and healthcare reimbursement programs must be improved. Without action, this poses a high probability to culminate in a deadly post-antibiotic era. This review will highlight some of the global health challenges faced both today and in the future. Furthermore, the new Infectious Diseases Society of America (IDSA) guidelines for resistant Gram-negative pathogens will be discussed. This includes new antimicrobial agents which have gained or are likely to gain FDA approval. Emphasis will be placed on which human pathogens each of these agents cover, as well as how these new agents could be utilized in clinical practice.

## 1. Introduction

In 2010, the Infectious Diseases Society of America (IDSA) developed the “10 × 20” initiative to prompt pharmaceutical companies to develop 10 new antibiotics by 2020 [1]. As a result, over 14 new antibiotics have since gained Food and Drug Administration (FDA) approval [1,2,3]. Despite these advances, a growing concern for resistant pathogens remains an epidemiological focal point both domestically and internationally [4]. In 2019, the United States Centers for Disease Control and Prevention (CDC) issued an Antibiotic Resistance Threats Report that highlighted growing resistance in numerous fungal and bacterial pathogens [1]. Featured within this document, carbapenem-resistant *Enterobacterales* (CRE) and carbapenem-resistant *Acinetobacter* (CRAB) were classified as urgent threats, which are the highest-level global threats. In light of the growing concern for antimicrobial resistance, focus has been placed on the development of new anti-infective agents that cover extended-spectrum beta-lactamases (ESBL), CRE, CRAB, and Ambler class B beta-lactamase-producing bacteria (MBL) [2,4,5,6].

Requirements have been established by the CDC, Centers for Medicaid and Medicare Services (CMS), and The Joint Commission (TJC) to combat the global antimicrobial resistance (AMR) pandemic [7,8,9]. All hospitals within the United States (U.S.) are now required to report both antimicrobial use (AU) and AMR data to the National Healthcare Safety Network (NHSN). Additionally, the CDC now mandates that all U.S. hospital systems have a designated individual(s) (i.e., medical provider, pharmacist, or both) to lead all antimicrobial stewardship (AMS) efforts within the institution. These efforts should consist of developing and implementing AMS treatment guidelines/protocols, communicating and collaborating with medical staff, and providing competency-based training and education. Lastly, the CDC recommends that hospital systems provide adequate funding to AMS efforts to ensure all TJC and CMS standards are met [7,8,9].

As multi-drug-resistant (MDR) infection rates rise, the rapid diagnosis and treatment of resistant infectious processes are vital to ensure a higher probability of clinical success [10,11,12,13,14,15,16,17]. Historically, diagnostic workup and treatment of patients included medical examination by a licensed provider and determination to use antibiotics was informed solely by culture-driven selection of an antimicrobial agent [18]. However, today, antibiotic selection has become increasingly convoluted due to the widespread use of extended-spectrum Gram-negative agents [19,20]. For example, pathogens such as CRAB have extremely limited treatment options, with some being associated with dose-limiting toxicities [21,22,23]. To further complicate matters, not all institutions have rapid diagnostic tools at their disposal. Without the capability for internal rapid diagnostic testing, outside microbiological testing must be utilized therefore significantly delaying the time to diagnosis, and ultimately the time to targeted treatment [14,16,24,25,26,27,28]. Lastly, diagnostic stewardship has become a major focus in combating the increasing AMR crisis. Data have shown that inappropriate diagnostic testing can leading to both the unnecessary prescribing of antibiotics and delays in appropriate antibiotic therapy. Therefore, it is imperative that clinicians utilize the correct test in the appropriate clinical scenarios in order to avoid inappropriate prescribing of antibiotics and/or delays in targeted therapy [29,30,31,32].

This review will highlight the global AMR pandemic and will discuss the current IDSA MDR guidance documents as well as potential antimicrobial agents on the horizon.

## 2. New Guidance Documents Have Been Established

In 2023, the ISDA published updated guidance documents for the treatment of antimicrobial-resistant Gram-negative infections [23]. The major pathogens highlighted in this document were ESBL-producing *Enterobacterales* (ESBL-E), AmpC-producing *Enterobacterales* (Amp-C-E), CRE, *Pseudomonas aeruginosa* with difficult-to-treat resistance (DTR-P), CRAB, and *Stenotrophomonas maltophilia* (*S. maltophilia*). The treatment recommendations from the guidance documents will briefly be discussed further for ESBL-E, CRE, and CRAB.

### 2.1. ESBL-E

Oxyimino cephalosporins were introduced to the market in the early 1980s, and by the late 1980s/early 1990s, reports of ESBL-E began to emerge [33]. Once considered a healthcare-acquired infection, ESBL-E is now commonly seen in the community setting [34]. This shift has led to the global rise of infections involving ESBL-E, and genetic mobilization of resistance patterns has become a major focal point in epidemiological and global health programs. Without a solution to slow the rapid global dispersion of ESBL-E isolates, rates of infections involving these pathogens will continue to rise [17,33,35].

In 2007, Melzer and colleagues prospectively collected clinical and microbiological data on adult patients with *Escherichia coli* (*E. coli*) bacteremia. These authors determined that patients with *E. coli* bacteremia had increased mortality rates. They concluded that delays in the initiation of appropriate antibiotic therapy in these patients were associated with worse clinical outcomes including death [36]. This study highlights the importance of early recognition of infections involving ESBL-E, although this idea has been recently challenged without mirrored results [37]. Regardless of mortality risk, limitations such as inaccessibility to rapid diagnostics, continue to be a challenge for many healthcare systems globally. Therefore, many clinicians are left to rely on the use of common susceptibility patterns (carbapenem and cephamycin susceptibility generally remain intact) rather than highly specific genotypic data to guide therapy. Consequently, this practice may result in prolonged durations of non-targeted or inappropriately targeted antimicrobial therapy [12,38]. Due to growing rates of AMR, the implementation of rapid diagnostics and their potential benefit will remain a focal point in clinical practice [28,39,40,41,42,43].

Treatment of ESBL-E is dependent on the source of infection. Treatment approaches for cystitis can range from oral options such as fosfomycin to one-time doses of an aminoglycoside. Treatment options for infections outside of the bladder are limited, and most clinicians favor carbapenems, particularly for bacteremia. However, there is still debate among providers regarding the usage of fluoroquinolones and/or trimethoprim-sulfamethoxazole (TMP-SMX) to prevent carbapenem exposure [23]. For more details on ESBL-E, refer to Table 1.

### 2.2. CRE

CRE is classically defined by the CDC as an isolate demonstrating resistance to at least one of the carbapenem antibiotics (ertapenem, meropenem, doripenem, and/or imipenem) or producing a carbapenemase [44]. The discovery of CRE dates back to 1996, the year in which meropenem gained FDA approval for complicated skin and skin structure infections [45]. Numerous enzymes have emerged that elicit carbapenem resistance; however, *Klebsiella pneumoniae* carbapenemase (KPC) remains the major enzyme responsible for CRE in the U.S. [5,45]. Treatment of CRE has gained global attention for drug development and numerous agents have come to the market targeting CRE, including ceftazidime-avibactam (CTZ-AVI), meropenem-vaborbactam (MV), and imipenem/cilastatin/relebactam (ICR) [23,44]. However, Gram-negative coverage gaps remain as many of these newer agents failed to fill a niche clinical role [46]. Additionally, resistance to newer Gram-negative broad-spectrum antibiotics, such as CTZ-AVI, is increasing and few options are currently available to treat these resistant pathogens [47,48].

Detection of CRE presents many challenges, with the greatest being the lack of rapid antimicrobial susceptibility testing (AST) in many national institutions [49,50,51,52]. Given the delays in microbiological results in organizations without rapid AST, many patients are empirically placed on broad-spectrum antibiotics until susceptibility reports are obtained. This may be problematic as patients who do not presently have an infection involving an MDR pathogen can have both alterations in their gut microbiota and an increased risk of future MDR infections [53,54,55]. Conversely, patients with an acute infection involving an MDR pathogen (i.e., CRE) will have major delays in therapy which can result in worse clinical outcomes for the patient [12,56,57].

CRE treatment, like ESBL-E, is dependent on the source of infection. The treatment for cystitis can range from oral options such as TMP-SMX, fluoroquinolones, and fosfomycin. A one-time parenteral dose of an aminoglycoside is also an alternative option for cystitis. Treatment beyond cystitis has limited options, and most clinicians favor newer antimicrobial agents such as CTZ-AVI or MV, although fluoroquinolones and TMP-SMX may have clinical roles as well depending on the source of infection [23]. For more details on CRE, refer to Table 1.

### 2.3. CRAB

CRAB has historically been considered a low-virulence pathogen; however, over the last two decades, it has emerged as a major global threat, predominantly due to its increasing drug resistance [58,59]. The decision to treat CRAB is often centered around colonization versus contributing pathogen [59]. If treatment is warranted, limited options are available, most of which have dose-limiting toxicities [59,60]. The recent attention on CRAB treatment has led to the FDA approval of sulbactam-durlobactam for the treatment of pneumonia involving CRAB [61].

Detection of CRAB is often straightforward and commonly involves both culture and AST [23,62,63,64,65]. Once identified, additional susceptibility reports are often needed for agents not routinely tested via AST (i.e., cefiderocol, sulbactam-durlobactam, minocycline). The treatment of CRAB is dependent on the level of illness severity, with severely ill patients likely needing two or more agents with susceptibility to the CRAB isolate [23]. For respiratory tract infections, a high dose (27 g/day) of ampicillin-sulbactam and tetracyclines remain the focal point of therapy [23,60]. Additional options for respiratory tract infections include aminoglycosides, cefiderocol, polymyxin-B, sulbactam-durlobactam, and possibly fluoroquinolones. Of these options, cefiderocol and sulbactam-durlobactam have gained favor in clinical practice. This likely stems from their favorable side effect profile compared to the other agents listed [23,60,61,66]. However, clinical trials comparing sulbactam-durlobactam vs. cefiderocol for the treatment of CRAB have not been completed. Nonetheless, it is debatable if cefiderocol should be utilized as a first line agent for CRAB as data has surfaced demonstrating the development of cefiderocol resistance during treatment for infections involving CRAB [67,68,69]. Additionally, initial data from the CREDIBLE-CR trial was not in support of using cefiderocol over best available therapy (BAT), although subsequent data, including a meta-analysis involving the CREDIBLE-CR trial, has argued against this conclusion [70,71]. With sulbactam-durlobactam gaining favor as a first-line therapy in moderate to severe infections involving CRAB, the future usage of cefiderocol for this indication should be reserved for MDR infections [66,72].

For chronic wound infections, treatment options mirror those seen in respiratory tract infections, and shorter durations of therapy are preferred in most clinical scenarios [73]. However, as previously mentioned, colonization vs. true pathogen is often discussed in clinical practice, especially for non-sterile sites of infection [60,73]. For more details on CRAB, refer to Table 1.

## 3. Epidemiology of Bacterial Resistance in the U.S. and Globally

### 3.1. Ambler Classification

In order to understand the landscape of resistance, it is crucial to grasp the major mechanisms bacteria use to gain resistance. Mechanisms of resistance can be broadly placed into four categories: enzymatic inactivation, porin channel loss, target modification, and efflux pumps [74,75,76]. Although all four mechanisms are important, this review will primarily focus upon enzymatic reactions, specifically beta-lactamases.

Enzymatic inactivation, caused by beta-lactamases, can be functionally categorized via either the Ambler classification system or the Bush-Jacoby-Medeiros classification system [77,78,79,80,81]. For the purpose of simplification, the Ambler class will be discussed. The Ambler class is broken into four major classes: A–D. Beta-lactamases are divided into these four main classes based on their amino acid sequences and functional characteristics. Each class encompasses distinct mechanisms of resistance, substrate specificities, and clinical implications [78,80,81]. A summary table of the Ambler classification system can be seen in Table 2.

Class A beta-lactamases are often referred to as “penicillinases” and are commonly found in Gram-negative bacteria such as *E. coli* and *Klebsiella pneumoniae*. These enzymes predominantly hydrolyze penicillins and cephalosporins and are inhibited by clavulanic acid. Notable examples include the widely studied temoneria (TEM) and sulfhydryl reagent variable (SHV) enzymes, which played a significant role in the development of resistance to beta-lactam antibiotics [23,78,80,82].

Class B beta-lactamases, also known as metallo-beta-lactamases (MBLs), require divalent metal ions, specifically zinc, for their catalytic activity. Unlike other classes, class B enzymes are inhibited by metal chelators such as ethylenediaminetetraacetic acid (EDTA). MBLs are commonly associated with MDR Gram-negative pathogens, including *P. aeruginosa* and *Acinetobacter baumannii* (*A. baumannii*). Their ability to hydrolyze a broad range of beta-lactam antibiotics, including carbapenems, poses a serious therapeutic challenge in clinical settings [23,78,80,82,83,84].

Class C beta-lactamases, or cephalosporinases, exhibit a broad substrate profile, hydrolyzing cephalosporins and penicillins. These enzymes are often chromosomally encoded and contribute to resistance in Enterobacteriaceae such as *Enterobacter cloacae* and *Citrobacter freundii*. Additionally, some penicillins and cephalosporins are strong inducers of Class C beta-lactamases, specifically AmpC, and can lead to phenotypic changes post-exposure. Due to this inducible nature, beta-lactams that are both strong inducers of AmpC and strong substrates are often avoided in practice to prevent phenotypic changes that can render certain beta-lactams ineffective. Lastly, class C beta-lactamases are not inhibited by clavulanic acid but can be inhibited by certain BLIs such as tazobactam. However, in clinical practice, cefepime, carbapenems, and fluoroquinolones have become mainstays of treatment for pathogens thought to harbor AmpC [23,78,80,82,85,86].

Class D beta-lactamases, also known as oxacillinases (OXA), primarily hydrolyze oxacillin and cloxacillin, conferring resistance to penicillins and cephalosporins. They are commonly found in Gram-negative pathogens like *Acinetobacter* spp. and *Pseudomonas aeruginosa* (*P. aeruginosa*). Class D enzymes are often associated with intrinsic resistance in these organisms and contribute to the challenge of treating infections caused by MDR strains. Furthermore, in the United States, OXA-48 has become a major concern for the development of CRE, and few treatment options are currently available that specifically target OXA-48 [23,80,87,88].

### 3.2. How Does Global Resistance Occur and Spread

The burden of AMR has brought upon the need for global One Health perspectives [89,90]. The concept of One Heath involves human health, animal health and environmental determinants at the local, national, and global levels to understand the complex interactions between them. By understanding these complex relationships, and their interdependency, approaches can be taken to optimize the health of people, animals, and ecosystems [90]. This tactic is vital when evaluating opportunities to slow the spread of MDR pathogens as AMR is a multifaceted process involving human medicine, wildlife health, environmental health, and health economics [1,90,91]. Data have demonstrated that AMR is a complex issue that involves overprescribing and overutilization of antibiotics in both humans and animals, and without a long-term shift towards eliminating unnecessary antibiotic usage in both, AMR rates will continue to rise globally [92,93].

To illuminate the influence of antibiotic use upon the global bacterial resistome, the animal–human–environment interface is crucial to investigate [92,93]. Notably, antimicrobials have a wide array of uses within plants and animals (i.e., domestic pets, livestock, fish hatcheries, and bee hives) in addition to human applications [93,94]. Many antimicrobial drug classes used for human populations are also prescribed for animals, which includes important human medicinal classes such as fluoroquinolones or broad-spectrum beta-lactams [92]. Additionally, persistence of antibiotic residues may be seen in wastewater treatment plants, livestock or wildlife waste, coastal waters, soil, and other environmental sources [95,96,97]. This has created a high-pressure system and ultimately the selection of bacterial resistance [94].

A myriad of pathways exist for the environmental conferral of AMR genes [75,91,92,98,99].

These pathways range from resistant zoonotic bacteria in soil infecting fruits, vegetables, and plants to agricultural antimicrobial applications transmitting antibiotic-resistant fungi to humans [94]. Additionally, compromise of aquaculture water sources may lead to residual antimicrobial compounds via fish products or excreta of fish, culminating in rapid spread leading to selective pressure for AMR genes [94,100,101]. Regardless of the source, it is imperative that environmental origins of resistance be identified so solutions can be created and mandated to slow the spread of AMR. This will likely require cumulative collaborative efforts among multiple experts including veterinarians, physicians, allied health professionals, and laboratorians. Additionally, tackling this global human health crisis will require establishing strong strategic partnerships between all nation-states with a targeted approach to prevent and control zoonotic and emerging infectious diseases [89,90,92,93,94].

### 3.3. The Burden of Global Resistance

Based on predictive statistical modeling across 204 countries and territories in 2019, an estimated 4.95 million deaths were associated with bacterial AMR [17]. Specifically, approximately 1.27 million deaths were directly attributable to bacterial AMR [17]. Within high-income countries (HICs) using the U.S. as an example, 60,813 (95% UI: 32,520–102,231) deaths were associated with bacterial AMR, and 14,987 (95% UI: 7712–25,156) deaths were attributable to AMR in 2019 [102]. *Staphylococcus aureus* (*S. aureus*) and *E. coli* were incriminated for most of these deaths with a high degree of resistance found among multiple antibiotic classes (up to 50% resistance associated with macrolides; 38% attributed to fluoroquinolones) [102]. Within low-income countries (LMICs), using Mali as an example, 7100 deaths were attributable to AMR with 29,700 deaths associated with AMR in 2019 [103]. Notably, mortality from AMR in Mali is higher than deaths from nutritional deficiencies, enteric infections, tuberculosis and respiratory infections, neglected tropical diseases and malaria, and cardiovascular diseases [104].

Both HICs and LMICs contribute to the global antibiotic resistance profile among bacterial populations colonizing humans, pets, livestock, and/or wildlife in various ways [105,106]. Additionally, AMR comes with negative implications and multiple studies have reported on the increased morbidity and mortality related to bacterial AMR within HICs and LMICs [107,108,109]. Moreover, specific bacterial species have been implicated as frequently causing pathology in humans—these are termed ‘ESKAPE’ pathogens (i.e., *Enterococcus* spp., *S. aureus*, *K. pneumoniae*, *A. baumannii*, *P. aeruginosa*, and *Enterobacter* spp.) [110,111,112]. Particularly concerning within LMICs are ‘MDR-ESKAPE’ pathogens. These pathogens have been cited as the primary source of morbidity and mortality among bloodstream infections in hospital settings [113]. Lastly, AMR bacteria, particularly MDR-ESKAPE pathogens, have been identified as high-risk indicators for increased economic costs among both LMICs and HICs [113]. This was emphasized in 2017 when The World Bank models estimated that a high burden of AMR could raise heath cost over one trillion dollars, highlighting the potential economic shortcomings that could arise if AMR rates are not slowed dramatically [114].

In 2024, the World Health Organization (WHO) released a bacterial priority pathogen report highlighting resistant trends for specific pathogens. Results from this report mirror those above. Specifically, carbapenem-resistant (CR) *Klebsiella pneumonia* and third generation cephalosporin-resistant (3GCR) *Escherichia coli* were both labeled as a level five threat, which is the highest level given for global resistant dispersion trends. Additionally, global resistance levels were highest for *Klebsiella pneumonia*, with over 30% of the isolates globally being labeled as CRE. Similar results were seen for CR *Escherichia coli* and 3GCR *Klebsiella pneumonia*, although overall resistance percentage levels were lower. Lastly, resistant pathogens per million people was highest with 3GCR *Escherichia coli* (>10,000 cases); however, the number of cases per million people for CR *Escherichia coli* and 3GCR *Klebsiella pneumonia* were above 5000. Together, these results illustrate the growing global AMR crisis [115].

### 3.4. Future Global Challenges with AMR

The global epidemiology of AMR presents a complex and evolving challenge to public health systems worldwide [17,116,117]. As previously discussed, the overuse and/or misuse of antimicrobial agents in human health, animal agriculture, and the environment have fueled the emergence and spread of resistant pathogens [91,118,119]. This phenomenon is exacerbated by factors such as inadequate infection prevention and control measures, poor access to clean water and sanitation facilities, and the globalization of travel and trade [91,118]. Consequently, AMR has become a pressing concern across diverse geographic regions and socioeconomic settings threatening the effectiveness of currently employed antibiotics [117].

Several key trends characterize the global epidemiology of AMR. To begin with, MDR pathogens, which exhibit resistance to multiple classes of antimicrobial agents, are increasingly prevalent and pose a significant clinical challenge [4,17]. Examples include MDR strains of *Mycobacterium tuberculosis*, methicillin-resistant *Staphylococcus aureus* (MRSA), CRE, CRAB, and MBL-producing pathogens [17]. Secondly, AMR disproportionately affects vulnerable populations (i.e., children, the elderly, and individuals with underlying health conditions) leading to higher rates of morbidity, mortality, and healthcare-associated infections [17,105,106,117,120,121,122]. Lastly, the emergence of extensively drug-resistant (XDR) and pan drug-resistant (PDR) strains represents a critical threat to global public health preparedness and response efforts [123,124].

Moving forward, targeted efforts to combat AMR should include supporting programs involved in the implementation of comprehensive AMS programs to promote the judicious use of antibiotics in healthcare settings, enhancing surveillance systems to monitor the spread of resistant pathogens and the identification of emerging resistance patterns, and investing in research and development of new antimicrobial agents and alternative treatment modalities [10,42,91,117,118,124,125,126,127,128,129,130,131,132,133]. Additionally, strengthening healthcare infrastructure, improving access to vaccines and basic healthcare services, and promoting interdisciplinary collaboration and international cooperation are essential to mitigate the impact of AMR and safeguard the effectiveness of antimicrobial therapy for future generations [106,127,132,134,135,136]. Likewise, enhancing awareness on environmental factors driving AMR is critical, and every effort should be made to decrease unnecessary antibiotic usage to help prevent both the acquisition and spread of resistant pathogens [98,132,137,138].

## 4. The Future of Drug Development

The need for novel antibiotics has never been greater, but outdated research and design methods, scarce governmental incentives for antibiotic development, and drug manufacturers’ inability to meet profit margins for newly launched antibiotics have stunted the manufacturing of new antibiotics [125,126,128,131,139,140,141,142,143,144,145,146]. To further compound these issues, hospital reimbursement measures often fail to account for the cost of treating MDR pathogens [145,147,148,149]. This places strain on the hospital administration as a balance between the cost of therapy and patient care must be delicately weighed. Governmental standards have also placed a great emphasis on controlling the spread of resistant pathogens by requiring strict infection prevention and AMS practices in all hospital systems in the U.S. [9,150,151]. Countries outside the U.S. have also placed value in AMS practices, placing focus on antibiotic usage both environmentally and in clinical practice [118,152,153,154]. Ultimately, to combat both AMR and the lack of novel antibiotic development, the fractured relationships between pharmaceutical industry and hospital administration must become a discussion point among key decision makers. Without reimbursement reform, improved drug and research development, and adequately funded global health programs which focus on the prevention of global dispersion of MDR pathogens, the future of novel antibiotic development and utilization will become further jeopardized [91,93,130,145,155,156,157,158,159].

Discussed below are the recent antibiotics under development and/or recently FDA-approved for usage in clinical practice. Their coverage, major clinical trials, and potential scenarios for utilization in clinical practice will be discussed in detail. A review of the major phase III clinical trial data can be seen in Table 3 for many of the agents discussed. Additionally, a review of the spectrum of activity for most agents can be seen in Table 4.

### 4.1. Cefepime-Taniborbactam

Taniborbactam is a boric acid beta-lactamase inhibitor (BLI) that is structurally similar to vaborbactam but has a wider spectrum of inhibition due to enhanced pharmacokinetic (PK) parameters and unique side chain structure compared to vaborbactam [160]. Taniborbactam, unlike avibactam, has in vitro activity against all four Ambler classes of beta-lactamases, making it one of the broadest Gram-negative covering BLI to date [80,160,161]. Specifically, taniborbactam provides coverage against ESBL, CRE, MDR Pseudomonas, and MBL-producing pathogens, making taniborbactam one of the few BLI with activity against Ambler class B enzymes [80,160,161,162,163,164].

In 2024, Wagenlehner et al. published a phase III clinical trial (CERTAIN-1) evaluating the efficacy and safety of cefepime-taniborbactam in the treatment of complicated urinary tract infections (cUTIs) [165]. This trial demonstrated both non-inferiority and superiority of cefepime-taniborbactam compared to meropenem in terms of clinical cure rates and microbiological eradication of pathogens. Additionally, cefepime-taniborbactam had a favorable safety profile, with adverse events comparable to those observed with other antibiotics.

Cefepime-taniborbactam is a promising therapeutic option for the treatment of MDR Gram-negative bacterial infections. With favorable PK and pharmacodynamic (PD) characteristics, a well-defined dosing regimen, and demonstrated efficacy and safety in phase III clinical trials, cefepime-taniborbactam holds great promise for addressing the growing threat of antibiotic resistance in healthcare settings [166,167]. However, potential heteroresistance is a concern. In 2023, Abbott et al. highlighted a high occurrence of heteroresistance to cefepime-taniborbactam in 34 MBL-producing *Enterobacteriaceae* isolates [168]. This finding was concerning on two fronts. One, heteroresistance is difficult to detect with traditional AST, and two, heteroresistance is likely more widespread than initially believed and may contribute to “induced” selection of cefepime-taniborbactam resistance. Nonetheless, the broad Gram-negative coverage of cefepime-taniborbactam is highly appealing, particularly in locations with a high prevalence of a specific MBL, the New Delhi MBL (NDM).

In February 2024, the FDA rejected the new drug application (NDA) for cefepime-taniborbactam citing the need for additional manufacturing information. With an unknown timeline for approval, the race for the first FDA-approved beta-lactam-BLI combination to cover all four Ambler class enzymes continues.

### 4.2. Cefepime-Enmetazobactam

Enmetazobactam is a novel penicillanic acid sulfone BLI similar to the BLI tazobactam. The difference is the addition of a methyl group to the triazole moiety making enmetazobactam an extended-spectrum BLI with the ability to increase the potency of cefepime and restore its activity against Ambler Classes A, C, and D [169]. Further analysis of the in vitro activity of cefepime-enmetazobactam revealed that its activity against Ambler Classes C and D only seemed to improve when *Enterobacterales* isolates co-produced ESBLs [170]. This suggested that the expression of Class C and D beta-lactamases was severely downregulated by the ESBL gene, resulting in the phenotypic appearance of an ESBL alone.

The phase III, randomized, double-blind, active controlled trial (ALLIUM) evaluated the efficacy of cefepime-enmetazobactam for the treatment of cUTIs or acute pyelonephritis in adult patients. Patients were randomized to either cefepime-enmetazobactam or piperacillin-tazobactam for up to 14 days. Results from the study determined that among patients with cUTI or acute pyelonephritis caused by Gram-negative pathogens, cefepime-enmetazobactam was superior to piperacillin/tazobactam with respect to both clinical cure and microbiological eradication [171]. Additionally, cefepime-enmetazobactam was found to be highly tolerable among patients.

Cefepime-enmetazobactam was manufactured to serve as a carbapenem-sparing agent against organisms harboring ESBLs [172]. With cost being a driving factor for many hospital systems, especially those with limited formularies, along with the fact that cefepime-enmetazobactam offers little coverage against MBL and CRE pathogens, it is difficult to imagine a clinical scenario where cefepime-enmetazobactam will be preferred over current therapeutic options, at least initially. Despite this, cefepime-enmetazobactam is an alternative treatment for pathogens co-producing both ESBL and OXA-48.

In February 2024, the FDA approved the usage of cefepime-enmatazobactam for cUTI in adults based off the data from the ALLIUM trial.

### 4.3. Cefepime-Zidebactam

Zidebactam belongs to the bicyclo-acyl-hydrazide class of beta-lactam enhancers, which is a derivative and a newer generation of the diazabicyclooctane (DBO) BLIs [173,174]. These newer generation DBO are considered dual-acting beta-lactam inhibitors and enhancers due to their ability to inhibit penicillin binding proteins (PBPs), enhancing the activity of an associated beta-lactam antibiotic that works on different PBPs, while also inhibiting activity against serine class A, C, and D beta-lactamases [173,174,175]. Zidebactam is a non-beta-lactam and thus, is not degraded by beta-lactamases. It has enhanced PBP2 binding in Gram-negative organisms, including *P. aeruginosa* and *A. baumanii,* and its enhancer effect is most demonstrated when combined with an agent targeted against PBP3 (i.e., cefepime) [173,176]. Cefepime-zidebactam has demonstrated in vitro activity against carbapenem-resistant *Enterobacterales*, *P. aeruginosa*, and some *A. baumanii*. Zidebactam alone has no activity against MBLs; however, when combined with cefepime, studies have demonstrated strong in vitro activity against these organisms [175,177,178,179,180]. Additionally, potential resistance mechanisms against cefepime-zidebactam are not fully described. However, based on in vitro data among the *Enterobacterales*, *K. pneumoniae* ST14 co-producing NDM and OXA-48-type carbapenemases were often found to be resistant. For *P. aeruginosa*, in vitro data suggest that resistance may be due to overexpression of efflux pumps; however, the extent of efflux pump activity was more pronounced with cefepime alone vs. in combination with zidebactam suggesting that zidebactam is not readily effluxed [176].

A phase III, non-inferiority clinical trial (NCT04979806) is currently underway evaluating the clinical efficacy and safety of cefepime-zidebactam for the treatment of cUTIs and pyelonephritis compared to meropenem. Until results of this trial are released, it is difficult to speculate about the potential role of zidebactam in clinical practice. However, the combination of cefepime and zidebactam has demonstrated activity against carbapenem-resistant Gram-negative organisms and is a potential option in the setting of MDR Gram-negative infections. Most notably, there have been two case reports highlighting the successful compassionate use of cefepime-zidebactam. In both case reports, cefepime-zidebactam was used as salvage therapy against NDM-producing *P. aeruginosa* in the setting of intra-abdominal infection and disseminated infection complicated by necrotizing ecthyma gangrenosum and respiratory infection [181,182].

### 4.4. Sulopenem

Like all beta-lactam antibiotics, sulopenem inhibits cell wall synthesis through binding to PBPs and inhibiting the final transpeptidation step of peptidoglycan synthesis. Specifically, sulopenem binds to the following PBPs with an order for affinity being greatest to least: PBP2, PBP1A, PBP1B, PBP4, PBP3, and PBP5 or 6 [183,184]. Structurally, sulopenem shares similarities and is often confused with carbapenems; however, carbapenems contain a proline ring while penems contain a thiazoline ring that produces a smaller bond angle, reducing stress on the beta-lactam ring, and protects against enzymatic degradation. Sulopenem has been shown to have in vitro activity against ESBL and AmpC-producing *Enterobacterales* [185]. Additionally, its Gram-positive and anaerobic activity seems to mimic other carbapenems such as meropenem and imipenem, including minimal activity against *Enterococcus faecalis* [186]. Sulopenem is unaffected by many beta-lactamases with a few exceptions. MBLs and carbapenemase-producing organisms are resistant to sulopenem. Furthermore, sulopenem is ineffective against other resistance mechanisms such as efflux pumps and porin channel changes [187].

Sulopenem has been developed in both intravenous and oral formulations. The PK properties of intravenous sulopenem are similar to that of other carbapenems. Early studies showed that the oral prodrug formulation of sulopenem had variable bioavailability ranging from 20–34% [188]. Further studies revealed that bioavailability increases by 23.6% when given with food alone and up to 62% when administered with food and 500 mg of probenecid. It was noted that the effects of probenecid on bioavailability were greater when given with food. There was a difference of a 7.3% increase when sulopenem/probenecid were given alone and a 40.7% increase when both medications were given in combination with food [189].

Two major trials for sulopenem have been completed to date. The first trial, SURE-1, evaluated sulopenem as a treatment option for uncomplicated UTIs caused by Gram-negative bacteria [190,191]. In the second trial, SURE-2, evaluated sulopenem as a treatment option for cUTIs and acute pyelonephritis caused by Gram-negative bacteria [192,193]. The results of these trials demonstrated that sulopenem was non-inferior to standard antibiotics in treating both uncomplicated and complicated UTIs, indicating its potential as an alternative treatment option. In addition, sulopenem demonstrated a favorable safety profile with few adverse effects reported, including no increases in the incidence of *Clostridioides difficile* (*C. difficile*) colitis.

### 4.5. Aztreonam-Avibactam

Aztreonam is a monobactam, working similarly to others in its class by binding to PBPs and inhibiting cell wall synthesis. Aztreonam can withstand hydrolyzation from MBLs, unlike other beta-lactam antibiotics. Despite its activity against MBL producers, these isolates often co-produce serine-beta-lactamases (AmpC beta-lactamases, ESBLs, and KPCs) which can hydrolyze it. Avibactam is a DBO non-beta-lactam BLI with activity against Ambler class A, C, and some class D beta- lactamases [194]. In combination with avibactam, the degradation of aztreonam is prevented as the avibactam component inhibits these co-produced beta-lactamase enzymes. Previous studies have cited the combination of CTZ-AVI and aztreonam as a treatment option for serious infections involving MBL producers [195,196]. Therefore, a combination product of aztreonam-avibactam proves to be a promising antimicrobial agent. Aztreonam/avibactam’s spectrum activity includes coverage against carbapenemase-producing *Enterobacterales* including those producing KPCs, VIM, IMP, NDM, and OXA-48. Additionally, the combination has activity against *Pseudomonas aeruginosa* and *S. maltophilia*. Aztreonam/avibactam lacks activity against *A. baumanii* due to OXA-type enzymes [197,198,199]. Pseudomonal resistance to aztreonam/avibactam has been attributed to production of *Pseudomonas*-derived cephalosporinase 1 (PDC), OXA enzymes (not including OXA-48), loss of porins, and overexpression of efflux pumps [197]. Resistance to *Enterobacterales* is suspected to be attributed to amino acid insertion in the PBP3 determinants, reducing aztreonam’s ability to bind [197].

The phase IIa open-label, multicenter study REJUVENATE studied the PK profile, safety, and efficacy of aztreonam/avibactam in patients with complicated intra-abdominal infections (cIAIs). The study supported the use of aztreonam/avibactam 500/167 mg loading dose infused over 30 min followed by 1500/500 mg every six hours maintenance regimen with doses infused over three hours. Dosing was determined for patients with an estimated creatinine clearance > 50 mL/min [200]. The REJUVENATE trial cited the most common adverse reaction to be an increase in hepatic enzymes, occurring in 26.5% of patients, most of which were asymptomatic and recovered upon discontinuation of treatment. Diarrhea was listed as the second most common adverse event, but none associated with *C. difficile* [200].

In clinical practice, aztreonam-avibactam holds promise as a combination therapy with coverage against all four Ambler beta-lactamase classes. Specifically, this combination may prove to be useful in combating pathogens co-harboring both MBL and class A/D enzymes.

### 4.6. Sulbactam-Durlobactam

Sulbactam is a well-known BLI with antibacterial activity against *A. baumannii* [23,201,202]. However, resistance to ampicillin/sulbactam is growing, leaving a major coverage gap in clinical practice for CRAB [17,203,204,205]. To combat this, sulbactam was commercially launched with durlobactam, which is a DBO BLI with activity against class A, C, and D beta-lactamases [206,207,208]. With structural similarities to avibactam, its endocyclic double bond and methyl substituent enhance the potency of durlobactam as a BLI and allow the inhibition of a wide range of Class D beta-lactamases commonly produced by *A. baumannii* [209]. Of note, durlobactam has been reported to have intrinsic activity against some species of *Enterobacteriaceae* through inhibition of PBP2, but it does not have intrinsic activity against *A. baumannii* when given on its own; however, both in vivo and in vitro activity have been achieved when given in combination with sulbactam [210,211].

In 2023, the ATTACK trial was published evaluating the usage of sulbactam-durlobactam in patients with either hospital-acquired pneumonia (HAP), ventilator-associated pneumonia (VAP), and/or bacteremia caused by *Acinetobacter baumannii-calcoaceticus* complex (ABC). Sulbactam-durlobactam was compared to colistin, and all patients received imipenem-cilastatin as background therapy. Results from this trial demonstrated that sulbactam-durlobactam was non-inferior to colistin for the primary endpoint of all-cause 28-day mortality. Additionally, sulbactam-durlobactam was less nephrotoxic compared to colistin [206].

Results from the ATTACK trial led to the FDA approval of sulbactam-durlobactam for HAP/VAP caused by susceptible isolates of ABC in 2023. Given the rise in incidence of CRAB cases in the U.S., sulbactam/durlobactam has potential for use in other MDR CRAB infections outside of the respiratory tract, including those resistant to other salvage therapy options [212].

## 5. Other BLI/BL/BLI Combinations

### 5.1. Xeruborbactam

Xeruborbactam (QPX7728) is a cyclic boronate inhibitor that is active against all four Ambler beta-lactamase classes. Xeruborbactam has shown in vitro activity against MBL isolates not inhibited by taniborbactam, specifically IMP and NDM-9 [213,214,215]. Additionally, xeruborbactam has demonstrated the ability, in vitro, to recover meropenem susceptibilities [213,215,216]. In a study by Lomovskaya et al., xeruborbactam was able to increase potency against meropenem-resistant KPC-producing strains of *K. pneumoniae*, NDM-1-producing strain of *E. coli*, and VIM-1-producing strain of *K. pneumoniae* [214]. Together, these results suggest xeruborbactam may have a role as both a recovery agent for certain beta-lactam antibiotics and a treatment option for MBL-producing bacteria.

Current phase III studies are lacking for xeruborbactam. However, multiple phase I trials have been completed. These trials demonstrated a favorable safety and PK profile for xeruborbactam [217,218]. Additional clinical data are needed to determine the role of xeruborbactam in clinical practice, but in vitro data favor further exploration of xeruborbactam.

### 5.2. Nacubactam

Nacubactam (OP0595, RG6080) is a DBO BLI with a distinctive dual mechanism of action compared to its sister compound avibactam [219,220,221]. It demonstrates activity against a broad spectrum of beta-lactamases, including class A, C, and some class D enzymes. Additionally, nacubactam is also an inhibitor of PBP2 in the cell wall of *Enterobacteriaceae*, which enhances the activity of co-administered beta-lactams. Together, this broad spectrum of activity suggests potential efficacy against a wide range of MDR bacteria, including those producing ESBLs and CRE [219,220,221,222].

While there is limited published clinical trial data available, there are ongoing studies evaluating the efficacy and safety of nacubactam in combination with various beta-lactam antibiotics. A phase II trial published in *Clinical Infectious Diseases* in 2020, evaluated the efficacy of nacubactam in combination with meropenem for the treatment of cUTIs and cIAIs caused by CRE [219]. The results demonstrated favorable clinical outcomes and tolerability, suggesting the potential utility of nacubactam in this patient population. Two other phase III trials are actively recruiting to evaluate the efficacy of cefepime-nacubactam and aztreonam-nacubactam for cUTIs and infections involving CRE (Integral-1 and Integral-2 trials).

## 6. Conclusions

In 1928, Alexander Fleming discovered that *Penicillium notatum* inhibited *Staphylococcus* spp. within a Petri dish, and by the early 1950s, penicillin became a mainstay of treatment for numerous infections [223,224]. Unfortunately, upon its utilization in clinical practice, a biological time clock started for the global dispersion of penicillin-resistant pathogens. Fast-forward to today, and that same biological clock continues to tick for antibiotics deployed in clinical practice. Traditionally, researchers and clinicians have strived for the development of newer antimicrobial agents that have the potential to overcome emerging resistant mechanisms of commonly encountered pathogens, but this retrospective approach is not sustainable [146,225,226]. As AMR continues to emerge, and few novel antibiotics are on the horizon, the focus must shift to preventive measures to slow the spread of resistant pathogens [227,228,229]. These measures should consist of a combination of standardized AMS programs that are adequately funded, guideline and culture-driven antibiotic prescribing practices, especially in the outpatient setting, properly funded global health programs, and strict infection prevention policies that focus on the importance of hand hygiene and personal protective equipment [8,93,227,229,230,231,232]. Ultimately, without drastic changes in healthcare reimbursement, global awareness of AMR, and antibiotic research drug development, preventive and supportive measures will become the standard of practice for infections caused by resistant bacterial pathogens that were once susceptible to employed antibiotics. This generated environment will likely become the largest global pandemic in modern time.

## Figures and Tables

**Table 1:** IDSA Pathogen and Drug Summary.

	ESBL-E	CRE	CRAB	DTR Pseudomonas
Common Pathogens Harboring Beta-lactamase	*Escherichia coli*, *Klebsiella pneumoniae*, *Klebsiella oxytoca*, *Proteus mirabilis*	*Klebsiella pneumoniae, Escherichia coli*, *Proteus mirabilis*	*Acinetobacter baumannii*	*Pseudomonas aeruginosa*
CDC Threat Level	Serious	Urgent	Urgent	Serious
Common Phenotype	Resistant to ceftriaxone	Resistant to meropenem or ertapenem	Resistant to carbapenems	Resistant to Psa covering cephalosporins and/or carbapenems
Common Genotype (Ambler Class)	CTX-M-15, GES-1, SHV-2 (A)	KPC-2, IMI-1, SME-1 (A/D)	OXA-48, OXA-51, OXA-23/24 (A/C/D)	AmpC, OXA-10, VIM, GES (A/B/C/D)
Cystitis Treatment (mild infection)	Oral (PO) Nitrofurantoin TMP-SMX FQ (ciprofloxacin, levofloxacin) Fosfomycin (*E. coli* only) Intravenous (IV) Carbapenem (ertapenem, meropenem, imipenem-cilastatin) Single dose of aminoglycoside (tobramycin or gentamicin)	Oral (PO) Nitrofurantoin TMP-SMX FQ (ciprofloxacin, levofloxacin) Intravenous (IV) Single dose of aminoglycoside (tobramycin or gentamicin) (CR/MDR infection) Ceftazidime-avibactam (CR infection) Meropenem-vaborbactam (CR infection) Imipenem-cilastatin-relebactam (CR infection) Cefiderocol (CR/MBL/MDR infection) Colistin/polymyxin B (MDR infection)	Intravenous (IV) High dose ampicillin-sulbactam (up to 27 g/day) Sulbactam-durlobactam Cefiderocol (MDR/MBL)	Oral (PO) FQ (ciprofloxacin, levofloxacin) Intravenous (IV) Cefepime (not CR) Piperacillin-tazobactam (not CR) Ceftazidime (not CR) Carbapenem (meropenem, imipenem-cilastatin) Aztreonam (not CR/combination for MBL with avibactam) Aminoglycoside (tobramycin, amikacin) Ceftolozane-tazobactam (CR infection) Ceftazidime-avibactam (CR infection) Imipenem-cilastatin-relebactam (CR infection) Cefiderocol (CR/MBL/MDR infection) Colistin/polymyxin B (MDR infection)
Non-Cystitis Treatment (moderate-severe infection)	Oral (PO) TMP-SMX FQ (ciprofloxacin, levofloxacin) Intravenous (IV) Carbapenem (meropenem, imipenem-cilastatin, ertapenem) Aminoglycoside (tobramycin, gentamicin, amikacin) (CR/MDR infection) Ceftolozane-tazobactam (CR infection) Ceftazidime-avibactam (CR infection) Imipenem-cilastatin-relebactam (CR infection) Cefiderocol (CR/MBL/MDR infection) Eravacycline (cIAI) Colistin/polymyxin B (MDR infection)	Oral (PO) TMP-SMX FQ (ciprofloxacin, levofloxacin) Intravenous (IV) Ceftazidime-avibactam (CR infection) Meropenem-vaborbactam (CR infection) Imipenem-cilastatin (CR infection) Cefiderocol (CR/MBL/MDR) infection) Aminoglycosides (tobramycin, gentamicin, or amikacin) (CR/MDR infection)	Intravenous (IV) High dose ampicillin-sulbactam (up to 27 g/day) in combination with another active agent (cefiderocol, polymyxin B, minocycline, tigecycline) Sulbactam-durlobactam	Oral (PO) FQ (ciprofloxacin, levofloxacin) Intravenous (IV) Carbapenem (meropenem, imipenem-cilastatin) Aztreonam (not CR/MBL in combination with avibactam) Aminoglycoside (tobramycin, amikacin) Ceftolozane-tazobactam (CR infection) Ceftazidime-avibactam (CR infection) Imipenem-cilastatin-relebactam (CR infection) Cefiderocol (CR/MBL/MDR infection) Colistin/polymyxin B (MDR infection)

Abbreviations: ESBL-E (extended-spectrum beta-lactamase-producing *Enterobacterales*); cIAI (complicated intra-abdominal infection); CR (carbapenem-resistant); CRAB (carbapenem-resistant *Acinetobacter baumannii*); CRE (carbapenem-resistant *Enterobacterales*); CTX-M (cefotaxime-hydrolyzing beta-lactamase isolated in Munich); DTR (difficult-to-treat resistance); g/day (grams per day); GES (Guiana-Extended-Spectrum); IMI (imipenem-hydrolyzing beta-lactamase); IV (intravenously); KPC (*Klebsiella pneumoniae* carbapenemase); MBL (metallo-β-lactamase); MDR (multi-drug resistant); OXA (oxacillinase); PO (by mouth); Psa (Pseudomonas); SHV (sulfhydryl reagent variable); SME (*Serratia marcescens* enzymes); TMP-SMX (trimethoprim-sulfamethoxazole); VIM (verona integron-encoded metallo-β-lactamase).

**Table 2:** Ambler Classification System.

Class	Catalytic Center	Examples (Enzymes)	Substrate (Target)	Inhibited by (Treatment)
A	Serine	TEM-1, SHV-1 (penicillinases)	Penicillins	Amoxicillin-clavulanic acid Ampicillin-sulbactam Most cephalosporins
		CTX-M (ESBL)	Cephalosporins	Piperacillin-tazobactam (cystitis) Cefepime (cystitis) Carbapenems Fluroquinolones
		KPC (Carbapenemases)	Carbapenems	Ceftazidime-avibactam Meropenem-vaborbactam Imipenem-cilastatin-relebactam Cefiderocol Fluoroquinolones
B	Zinc	IMP, VIM, NDM	All beta-lactam antibiotics (not aztreonam)	Aztreonam + avibactam Cefiderocol Cefepime-taniborbactam (not FDA approved) Xeruborbactam (not FDA approved)
C	Serine	AmpC	Penicillins and most cephalosporins	Cefepime Carbapenems Fluroquinolones
D	Serine	OXA	Penicillins, cephalosporins, and carbapenems (depends on OXA subtype)	Amoxicillin-clavulanic acid Ampicillin-sulbactam Piperacillin-tazobactam Ceftazidime-avibactam (OXA-48) Cefiderocol (OXA-48) Fluoroquinolones

Abbreviations: CTX-M (cefotaxime-hydrolyzing beta-lactamase isolated in Munich), IMP (active on imipenem) (imipenem-hydrolyzing beta-lactamase); KPC (Klebsiella pneumoniae carbapenemase); NDM (New Delhi metallo-beta-lactamase); OXA (oxacillinase), SHV (Sulfhydryl reagent variable); TEM (Temoneira); VIM (Verona integron-encoded metal-beta-lactamase).

**Table 3:** Phase III trials of New Antimicrobial Agents.

Drug	Trial Name	Intervention	Source of Infection	Primary Endpoint	Results	Conclusion
**CEF-TANI**	CERTAIN-1	CEF-TANI (2 g + 0.5 g) or meropenem (1 g) q8h	cUTI	Microbiologic and clinical success on trial days 19 to 23 in the microITT population	70.6% in the CEF-TANI group and 58.0% in the meropenem group (treatment difference, 12.6 percentage points; 95% CI, 3.1 to 22.2; *p* = 0.009)	CEF-TANI was superior to meropenem for the treatment of cUTI
**CEF-TANI**	CERTAIN-2	CEF-TANI (2 g + 0.5 g) or meropenem (1 g) q8h	HAP/VAP	28-day all-cause mortality in the ITT population	Pending	Pending
**CEF-EM**	ALLIUM	CEF-EM (2 g + 0.5 g) or 4.5 g piperacillin-tazobactam q8h	cUTI or AP	Overall treatment success (clinical cure combined with microbiological eradication of infection)	79.1% in the CEF-EM group and 58.9% in the piperacillin-tazobactam group, (treatment difference, 21.2 percentage points; 95% CI, 14.3 to 27.9)	CEF-EM was non-inferior to piperacillin-tazobactam for the treatment of cUTI or AP
**CEF-ZIDE**	NCT04979806	CEF-ZIDE 3 g (2 g + 1 g) q8h or meropenem 1 g q8h	cUTI or AP	TOC for cUTI or AP at day 17 +/− 2 days	Pending	Pending
**SUL-DUR**	ATTACK	SUL-DUR (1 g each component) q6h or colistin 2.5 mg/kg q12h All patients received imipenem-cilastatin 1 g each component q6h	HAP/VAP, or BSI	28-day all-cause mortality the mMITT population NI margin was set at upper bound 95% CI of less than 20%	19% in the sul-dur group and 32% in the colistin group, (treatment difference of −13.2 percentage points; 95% CI, −30.0 to 3.5)	SUL-DUR was non-inferior to colistin, when each are given in combination with imipenem-cilastatin for the treatment of HAP, VAP, or BSI
**Sulopenem**	SURE 1	Sulopenem 500 mg/probenecid 500 mg PO twice daily for 5 days or ciprofloxacin 250 mg PO twice daily	Uncomplicated UTI	Combined clinical and microbiological response at day 12 in the mMITT population	65.6% in the sulopenem group and 67.9% in the ciprofloxacin group, (treatment difference −2.3 percentage points; 95% CI, −7.9 to 3.3)	Sulopenem was non-inferior to ciprofloxacin for the treatment of uncomplicated UTI
	SURE 2	Sulopenem 1000 mg IV once daily followed by oral sulopenem 500 mg/probenecid 500 mg or ertapenem 1000 mg IV once daily followed by oral ciprofloxacin 500 mg or amox/clav 875 mg twice daily	cUTI	Composite clinical and microbiologic outcomes at TOC in the mMITT population	67.8% in the sulopenem group and 73.9% in the ertapenem group, (treatment difference of −6.1 percentage points; 95% CI, −12 to −0.1)	Non-inferiority was not achieved by the sulopenem group for the treatment of cUTI
**AZT-AVI**	REVISIT	AZT-AVI (loading, extended loading and maintenance doses) ± MTZ 500 mg IV q 8 h vs. meropenem 1 g q 8 h ± colistin 9 million IU IV loading dose, followed by 9 million IU given IV daily in 2 or 3 divided doses	cIAI or HAP/VAP due to Gram-negative bacteria, including MBL-producing organisms	Clinical cure at TOC visit in ITT and clinically evaluable analysis sets	ITT: 68.4% in the AZT-AVI ± MTZ group and 65.7% in the meropenem ± colistin Clinically evaluable analysis set: 77% in the AZT-AVI ± MTZ group and 74.3% in the meropenem ± colistin group	AZT-AVI ± MTZ displayed similar efficacy compared to meropenem ± colistin for the treatment of cIAI and HAP/VAP
	ASSEMBLE	AZT-AVI (loading, extended loading and maintenance doses) ± MTZ 500 mg IV q 8 h or BAT	cIAI, HAP/VAP, cUTI or BSI with MBL Gram-negative bacteria isolated within 7 days prior to screening	Clinical cure at TOC in Micro-ITT analysis set at day 28	41.7% in the AZT-AVI ± MTZ group and 0% in the BAT group	Enrollment terminated early due to limited numbers of MBL associated infections (n = 15), no conclusions drawn from study results

Abbreviations: Amox/clav (amoxicillin/clavulanic acid); AP (acute pyelonephritis); AZT-AVI (Aztreonam-avibactam); BAT (best available therapy); BSI (bloodstream infection); CEM-EM (cefepime-enmetazobactam); CEF-TANI (cefepime–taniborbactam); CEF-ZIDE (cefepime-zidebactam); CI (confidence interval); cIAI (complicated intra-abdominal infection); cUTI (complicated urinary tract infection); HAP (hospital-acquired pneumonia); ITT (intention-to-treat); IU (international units); IV (intravenous); MBL (metallo-β-lactamase); Micro-ITT (microbiology intention-to-treat); MITT (modified intention-to-treat); mMITT (microbiologically modified intention-to-treat); MTZ (metronidazole); NI (non-inferiority); PO (by mouth); q (every); qh (every hour); SUL-DUR (sulbactam-durlobactam); TOC (test-of-cure); UTI (urinary tract infection); VAP (ventilator-associated pneumonia).

**Table 4:** Spectrum of Activity.

Ambler Class	CEF-TANI	CEF-EM	CEF-ZIDE	SUL-DUR	Sulopenem	AZT-AVI
**Class A:**						
CTX-M	✓	✓	✓	✓	✓	✓
KPC	✓	✓	✓	✓	—	✓
**Class B:**						
NDM	✓	—	✓	—	—	✓
VIM	✓	—	✓	—	—	✓
IMP	—	—	✓	—	—	✓
**Class C:**						
AmpC	✓	✓	✓	✓	✓	✓
**Class D:**						
OXA-48	✓	✓	✓	✓	—	✓
**Pathogens of Interest**						
**CRE**	✓	✓	✓	—	—	✓
**DTR** **Pseudomonas**	✓	✓	✓	—	—	✓
**CRAB**	—	—	—	✓	—	—
**Stenotrophomonas**	—	—	—	—	—	✓

Abbreviations: AZT-AVI (Aztreonam-avibactam); CEF-EM (cefepime-enmetazobactam); CEF-TANI (cefepime–taniborbactam); CEF-ZIDE (cefepime-zidebactam); CRAB (carbapenem-resistant *Acinetobacter baumannii*); CRE (carbapenem-resistant *Enterobacterales*); CTX-M (cefotaxime-hydrolyzing beta-lactamase isolated in Munich), DTR (difficult-to-treat resistance); IMP (active on imipenem) (imipenem-hydrolyzing beta-lactamase); KPC (*Klebsiella pneumoniae* carbapenemase); NDM (New Delhi metallo-beta-lactamase); OXA (oxacillinase), SUL-DUR (sulbactam-durlobactam); VIM (Verona integron-encoded metal-beta-lactamase).

## Data Availability

Not applicable.
